# Disembedding and re-embedding: the online interaction mechanisms of divorced youth in China

**DOI:** 10.3389/fpsyg.2024.1413129

**Published:** 2024-05-27

**Authors:** Junjie Wang, Jialiang Guo

**Affiliations:** School of Journalism and Communication, Shandong University, Jinan, China

**Keywords:** disembedding, re-embedding, divorced youth in China, dating apps, online interaction

## Abstract

**Introduction:**

In recent years, China’s divorce rates have remained high, especially in metropolitan areas such as Beijing and Shanghai, where rates reach up to 40%. Additionally, there has been a notable shift towards younger demographics in divorce cases. In a society that highly values marital harmony, divorce is often seen as a cultural transgression. Anthony Giddens’ theory of disembedding and re-embedding provides a useful framework for understanding these changes. This study addresses a gap in literature by focusing on the online social interactions of divorced Chinese youth, exploring their use of dating apps for emotional support and social reconnections.

**Methods:**

This qualitative study employed semi-structured interviews with 19 divorced young adults in China who engaged with dating apps such as Momo, Tantan, and Soul. Participants were recruited via Douban and Xiaohongshu. The interviews, conducted through WeChat voice calls and Tencent Meetings, lasted 45-70 minutes each. Data was analyzed using Nvivo12 to understand the disembedding and re-embedding processes in their online interactions, exploring themes such as motivations, self-presentation, and the transition from online to offline engagements.

**Results and discussion:**

The findings reveal that these individuals face societal challenges, biases, and the residual effects of past marriages, leading them to seek refuge in online environments to avoid stigmatization. In digital spaces, they cautiously engage, revealing a lack of confidence through selective self-disclosure. Their goals range from forming same-sex and opposite-sex friendships to seeking new romantic relationships, indicating a nuanced approach to remarriage and challenging stereotypes of dating app users. Re-engaging online, they discover social support and a sense of community, which aids in regaining confidence post-divorce, underscoring the complex interplay between societal influences and individual adaptation strategies in the digital age. The study highlights the unique challenges faced by this demographic, including maintaining anonymity and dealing with societal prejudices. Future research should consider a broader age range and gender differences to provide a more comprehensive understanding of the online behaviors and experiences of divorced individuals.

## Introduction

At the beginning of 2024, the Ministry of Civil Affairs of the People’s Republic of China (2024) published a report titled “Civil Affairs Statistical Data for the Four Quarters of 2023.” According to the data, a total of 2.593 million couples completed their divorce registrations in the four quarters of 2023. Despite the observed decline, divorce figures remain alarmingly high, particularly in metropolitan areas such as Beijing and Shanghai, where the divorce rate among married couples is estimated to be as high as 40% (Fu and Wang, 2019). Additionally, Yang and Sun (2021) highlight a demographic shift in divorce cases toward younger populations, indicating a significant, youth-centric transformation in divorce trends. In China, a society that highly values harmony, divorce and out-of-wedlock births are viewed as severe cultural transgressions. Divorce is often perceived as socially and morally undesirable, regardless of the challenges faced by couples (Fu and Wang, 2019). Empirical studies utilizing cross-sectional data on marital status and well-being consistently show that divorced individuals report significantly lower levels of happiness compared to their married counterparts (Wade and Pevalin, 2004; Amato, 2010). Divorce reflects changes in an individual’s happiness index and is accompanied by continuous psychological shifts. Numerous studies have explored the mental breakdown associated with divorce and the individual’s subsequent recovery (Kitson and Morgan, 1990; Amato, 2000). China ranks among the countries with the highest number of dating app users (Curry, 2022). In this digital era, online dating platforms provide a unique avenue for divorced individuals, particularly young adults, to forge new social connections (Veel and Thylstrup, 2018). These platforms not only facilitate an escape from the stigmatization encountered in offline settings but also offer a means to quickly overcome real-life judgments and gain emotional support (Wang, 2019).

Over the past decade, digital dating has become commonplace, with 30% of Americans having used dating websites or applications (Anderson et al., 2020). In recent years, the user base of dating apps in China has continually expanded, approaching 650 million instances in 2020 (iiMedia Research, 2021). This indicates that dating apps, as a social platform for strangers, have become thoroughly embedded in people’s daily lives. Despite extensive research on dating apps, such as dating psychology (Finkel et al., 2012), self-presentation (Ranzini and Lutz, 2017), and online dating behaviors (Sharabi and Timmermans, 2021), studies have also examined online socializing among Saudi divorced women (Luppicini and Saleh, 2017), online dating in the middle-aged divorced demographic (Dwyer et al., 2021), and online interactions of divorced Chinese women (Kuang et al., 2022). These studies provide a foundational basis for this research. However, overall, there is a lack of focus on the Chinese context and the divorced youth demographic within existing literature. Even when Chinese divorced populations are considered, there is a notable absence of qualitative perspectives, which limits deep analysis of the psychological changes within this group. This oversight neglects their emotional needs and motivations in interactions post-relationship breakdown, as well as the process of these interactions. Therefore, given this background, focusing on the online interactions of divorced youth in China holds significant practical relevance. To address this research gap, our study conducted semi-structured interviews with 19 divorced Chinese youth in 2023. As divorce becomes more prevalent among younger populations, selecting divorced youth in China as the study subjects aligns with current social conditions. This study is grounded in Anthony Giddens’ concepts of “disembedding” and “re-embedding,” which suggest that individuals disengage from their existing social relations and form new ones through spatial mobility. It focuses on the specific practices of divorced Chinese youth in online interactions, aiming to gain insights into the deeper dynamics of their interactions and psychological states.

Accordingly, this paper formulates three critical research questions to guide our investigation:

1. What are the engagement patterns of divorced Chinese youth on dating apps?
2. How do these online interactions facilitate their transition from disembedding, re-embedding?
3. What are the implications of re-embedding for their real-life circumstances?

## Navigating new connections: exploring the dynamics of dating app usage among divorced individuals

The life course is marked by a sequence of pivotal events, among which divorce and widowhood are often identified as major disruptions (Miller and Rahe, 1997). Marriage functions as a social construct wherein individuals actively craft, maintain, and interpret their life narratives (Berger and Kellner, 1964). Under typical circumstances, an individual’s social network contracts after marriage, becoming increasingly intertwined with that of their spouse (Gerstel, 1988). However, the dissolution of a marriage precipitates the rapid loss of these intra-marital benefits, leading to profound changes in the social networks of the individuals involved. The previously stable social circles may become fragmented, resulting in a significant transformation in the social structures (Botterman et al., 2014).

Divorced individuals frequently find themselves at a critical juncture, necessitating a reassessment of their social networks. Friends within these networks may face loyalty conflicts, struggling to maintain connections with both former partners. Such disruptions can severely impact divorced individuals, hindering their ability to swiftly reestablish normal interpersonal relationships. This fragmentation often results in reduced social support, which can intensify feelings of isolation and psychological distress (Milardo, 1987). In the Chinese context, where divorce is stigmatized, divorced individuals may fear discrimination and thus might refrain from sharing their experiences with family, friends, and colleagues, exacerbating their isolation (Taylor et al., 2004). Given these challenges, it is crucial for divorced young adults in China to seek new avenues for socializing. This exploration can enable them to move beyond past marital experiences and broaden their social networks, offering vital support during transitional periods.

Following a divorce, individuals are freed from marital constraints, increasing their need to seek new romantic partners or dating companions (Milardo, 1987; Gerstel, 1988; Albeck and Kaydar, 2002). Dating apps, which are fundamentally based on principles of liking and matching, have evolved into a digital romance matchmaking system (Finkel et al., 2012). These platforms align well with the interpersonal needs of divorced individuals by providing a respite from the discomfort and awkwardness associated with offline social interactions (Sautter et al., 2010). Consequently, dating apps serve both public and private roles in the interpersonal engagements of divorced groups. The current mode of dating leverages algorithmic recommendation features, which have become central to the functioning of online dating platforms (Sharabi, 2022). Despite concerns about algorithmic matching such as privacy issues (Narr, 2022) and control over user information (Lutz et al., 2020), an increasing number of users are opting to form relationships via dating apps (Vogels and Mcclain, 2023). These applications employ advanced machine learning and big data analytics to predict user preferences and aesthetics (Fellizar, 2015), ensuring mutual attraction rather than unilateral approval (Pizzato et al., 2013). As users grow to trust these algorithmic matches and engage in self-disclosure, the probability of successful dating outcomes increases (Sharabi, 2021). People tend to believe that sharing personal information enables algorithms to better serve their needs (Kapsch, 2022) and consider these algorithms to be more accurate and objective than human judgment (Sundar, 2020). However, such algorithmic recommendations can restrict their ability to freely choose personalized content (Ytre-Arne and Moe, 2021). Yet, some research suggests that users can actively engage with and influence the outcomes of these recommendations, rather than merely accepting them passively (Wang, 2020; Hu, 2023; Hu and Zhan, 2023). The proliferation of digital technology has ushered in the nuanced concept of “liquid surveillance,” a decentralized mechanism in which individuals engage in relational networks, simultaneously serving as both observers and subjects of observation (Bauman and Lyon, 2013, pp. 14–18). The inherent anonymity and surveillance capabilities of online platforms afford a level of comfort to divorced individuals, mitigating the anxiety associated with face-to-face interactions (Rosenfeld et al., 2019). Features such as geolocation in dating apps not only inform users of others’ proximity but also foster a platformized environment in which users can discreetly explore others’ interests and activities (Albrechtslund, 2008; Duguay et al., 2022). However, while dating apps facilitate real-life connections, they also commodify users, exacerbating uncertainties in the selection process (Bandinelli and Gandini, 2022; Narr, 2022). Consequently, users of dating apps are impelled to construct an “ideal self,” constantly negotiating the balance between the need for positive impression management and the desire to present their authentic selves (Ellison et al., 2006).

On dating apps, user self-presentation is influenced by a variety of motives. Chinese youth utilize dating apps with various objectives including curiosity, socializing, romance, and to navigate through post-breakup periods (Chan, 2020). This interest has led to the popularity of platforms like Tantan and Momo, despite criticisms that they promote casual hookups or one-night stands (Xu and Wu, 2019). Research among Shanghai’s online dating community identifies three distinct dating modalities: traditional dating, xiangqin (matchmaking), and a combination of both, reflecting the modern dating ethos of Chinese youth (Shen and Qian, 2022). Dating increasingly emphasizes individual desires and romantic intimacy, focusing not necessarily on marriage but on the process of developing emotional companionship. In contrast, arranged dating emphasizes matching conditions with marriage as the goal, often incorporating parental expectations and considerations of the traditional Chinese concept of “equally well married,” which involves aligning social and economic statuses.

In summary, substantial research has been conducted on social interactions through dating apps, which provides a solid foundation for this study. However, the current body of research has primarily focused on the mechanisms of platforms, purposes of interactions, and demographic profiles of users (e.g., Xu and Wu, 2019; Wu and Ward, 2020), as well as digital inequality and stigma interventions (Chan, 2018; Liu et al., 2022). Despite this extensive coverage, there remains a lack of exploration into the specific interpersonal interactions of divorced groups and the consequential changes in their lives and psychological states that these interactions facilitate.

## From disembedding to re-embedding: investigating the spatial mobility of social groups

Anthony Giddens introduced the concept of “disembedding” in his seminal work “The Consequences of Modernity,” where he defines it as the extraction of social relations from local contexts and their restructuring across extensive time–space dimensions (Giddens, 1991, p. 21). Following disembedding, individuals often feel a reduced connection to their original communities. This phenomenon is particularly evident among divorced young adults in China, who seek to sever ties with their previous community relationships to avoid emotional distress and societal stereotypes, and to actively reconstruct their psychological identities. Empirical data from various studies indicate that re-embedding is possible during the process of disembedding (Lee, 2011). As Giddens explains the concept of “re-embedding,” it involves “the re-transference or reconstruction of disembedded social relations so that they fit, whether on a partial or temporary basis, into the locational and temporal circumstances of locale” (Giddens, 1991, p. 79). Au-Yeung and Qiu (2022) observed confrontations between platforms and labor in the integration of gig workers into Hong Kong’s labor market. The internet, serving as a medium that facilitates the dissolution of ties between place and kinship, eases the maintenance of relationships over geographical distances, thereby offering possibilities for re-embedding. Liu and Wang (2022) focus on the social media empowerment and solidarity practices of Chinese truck drivers, who, through online mutual aid organizations and communities, have formed stratified networks of solidarity and mutual help. The internet facilitates this process by transcending traditional spatial and temporal boundaries, enabling these individuals to overcome geographical constraints and engage with disembedded social relationships online. While new intimate relationships begin to form through social support and emotional exchanges, potentially fostering new self-identities, these relationships often exhibit instability (Lee, 2005). Giddens (1992, p. 58) forwarded the notion of pure relationship – intimacy is based on mutual consent (vs. traditional social arrangement) and is vulnerable and subject to change, therefore, disembedding and re-embedding is common experience and a problem people have to deal with in modern society. In fact, dating apps facilitate the creation of an “intimacy network” that substantially increases users’ social capital. These networks, potentially oriented toward romantic engagement, enhance an individual’s capability to find a partner and establish a mutually satisfying relationship, thereby fostering the trend toward what Giddens describes as a “pure relationship.”

Georg Simmel posited that spatial mobility facilitates the formation of social relationships that would otherwise be constrained by physical or cultural distances. This mobility, as argued by Recchi and Flipo (2019), transforms the encounter of strangers into a microcosm of society. For instance, research by Sun and Lei (2017) demonstrates that urban residents, particularly those around 30 years old or younger and without siblings, often seek support beyond their immediate urban environments, such as in rural or small-town areas, thereby prompting cross-regional group mobility. Following the reinstatement of the college entrance examination in 1977, China has witnessed a peak in population movement. This migration is primarily driven by college students and migrant workers from villages and small towns who move to urban centers, becoming key agents of social mobility (Sun and Lei, 2017). However, these migrants often remain as strangers within urban settings, facing social exclusion from established cultural groups, which impedes their integration into the new community (Zhang, 2001). Simmel also explored the interplay between technological advancement and spatial mobility. According to Calabrese et al. (2011), while information technology has expanded the potential for long-distance interactions, most interpersonal communications still occur at close range. An illustrative case is provided by ‌Witmer (1997), who examined the world’s largest and most successful sobriety organization. His study highlighted its intricate social interaction system and demonstrated how members navigate the transition from disembedding to re-embedding within social contexts.

Recent research has characterized the described spatial mobility as daily mobility, exemplified by routine movements between home and the workplace (Masso et al., 2019). In the context of divorced young adults in China, this mobility extends into the digital realm. Having lost ties with their original communities, these individuals navigate into internet spaces to forge new identity affiliations and establish new communities within this spatially fluid environment. Inevitably, the process of re-embedding varies significantly across social groups, with individuals experiencing diverse objectives, relationships, sense of belonging, and attachment status. To encapsulate this variability, some scholars have introduced the concept of “differentiated embedding” (Ryan, 2018), which highlights the stratified and multifaceted nature of embedding. This term also acknowledges the existence of embedding inequality, suggesting that the process of integration into new social contexts is not uniform but rather layered and complex.

In conclusion, Giddens’ concepts of disembedding and re-embedding provide a robust theoretical framework for this study. However, the disembedding and re-embedding processes among Chinese divorced youth exhibit unique characteristics. Notably, the transition from physical to cyberspace involves no change in physical distance but encompasses virtual mobility within the context of the internet. This form of movement differs from traditional spatial mobility previously discussed. Secondly, this process is incomplete; they cannot fully sever ties with their real-world social relationships, resulting in a dual influence from both real and cyber spaces. While existing research has extensively explored the impact of traditional social structures and the internet society on individuals’ disembedding and re-embedding, less attention has been paid to the particular circumstances of divorced young adults in China. This demographic may leverage the potential to disembed from real spaces and re-embed into cyberspaces, facilitating access to social support and identity affirmation. Additionally, the life situations of these individuals post-re-embedding in China also warrant further examination.

## Methodology

To address the research questions outlined, this study utilizes a qualitative interview methodology, widely recognized for its effectiveness in exploring individual life experiences and interpreting socio-cultural phenomena (Brinkmann, 2013, pp. 47, 72). This approach is particularly suited for examining the nuanced contexts and personal experiences crucial to understanding the dynamic practices of online social interactions among divorced Chinese youth. We conduct semi-structured interviews to probe into their online social behaviors on dating apps, allowing for the collection of rich, in-depth insights. These insights are gleaned from participants’ narratives and responsive follow-up questions within a conversational setting. The interview framework for this research is structured around several key themes. Initially, it explores the disembedding experiences of divorced Chinese youth, investigating their post-divorce life circumstances, interactions with family and friends, changes in social networks and mindsets, and motivations for seeking new social environments. The study then shifts focus to the re-embedding process facilitated by online interactions, examining motivations, strategies, objectives, challenges, and self-presentation online, as well as the distinctions between online and offline interactions and the transition of online connections into real-world engagements. Lastly, the study assesses the post-reembedding life alterations experienced by these individuals, including their engagement with other divorced persons, perceptions of new social connections, receipt of social support, and expansion of their social networks.

Adhering to the principles of data saturation, accessibility, and purposive sampling (Merriam and Tisdell, 2015), this study recruited participants through the social media platforms Douban and Xiaohongshu. Douban, a social media website, and Xiaohongshu, an app, are both popular in China. The study involved 19 divorced young adults in China who had engaged with dating apps such as Momo, Tantan, and Soul for at least 1 month post-divorce. Interviews ranged from 45 to 70 min. These platforms are notably popular in China for dating and social interactions, with a significant daily user base. Momo is similar to Grinder, with its socialization mode primarily based on geographical location to find nearby users. Tantan is akin to Tinder, where friendship is initiated through swiping left or right on profile cards to express liking or disliking. Soul facilitates friendships through random matching. Douban, on the other hand, fosters friendships and interactions within interest communities and topic groups. Notably, some participants used Douban (Table 1). Though Douban is a general social networking site and is not only designed for online dating, the platform was actively used by some of our participants as a platform for online dating. To ensure a broad representation of experiences, the study utilized diversity sampling, capturing a wide array of variations among participants. The participant group comprised individuals aged between 25 and 40 years, with educational levels ranging from high school to graduate studies. The sample included 9 men and 10 women; three were in romantic relationships, while the others were single. Ten participants were parents, and nine were not. All participants identified as heterosexual. Recruitment materials emphasized the confidentiality of responses, with anonymity assured and reaffirmed prior to each interview. Each participant was fully briefed on the study’s aims and provided written consent before their interviews. Table 1 in the manuscript displays the demographic information of the participants at the time of their interviews.

**Table 1:** Participants’ demographic characteristics at the time of interview.

Subjects	Gender	Age	Education	Marital status	Dating app
S1	Male	35–40	Bachelor	Single	Momo, Tantan
S2	Male	25–30	Bachelor	Single	Momo, Tantan
S3	Female	25–30	Bachelor	Single	Soul
S4	Male	25–30	Bachelor	Single	Momo, Tantan
S5	Male	25–30	High School	Single	Soul, Tantan
S6	Female	30–35	Master	In a Relationship	Soul
S7	Female	30–35	High School	Single	Momo
S8	Male	35–40	Bachelor	Single	Douban
S9	Female	35–40	Bachelor	Single	Douban
S10	Female	30–35	Master	Single	Tantan
S11	Female	35–40	Master	Single	Momo, Soul
S12	Male	30–35	PHD	Single	Douban
S13	Female	35–40	High School	Single	Momo, Tantan
S14	Male	35–30	Bachelor	Single	Tantan
S15	Female	30–35	Bachelor	Single	Soul
S16	Male	25–30	Bachelor	In a Relationship	Tantan
S17	Female	30–35	Bachelor	Single	Qing Teng Zhi Lian
S18	Female	35–40	Master	In a Relationship	Douban, Soul
S19	Male	35–40	Bachelor	Single	Soul

The in-depth interviews were conducted using WeChat voice calls and Tencent Meetings. With the explicit consent of the interviewees, all sessions were recorded in their entirety. Following the completion of the interviews, the recordings were transcribed verbatim by the research team. For data analysis, the research team utilized Nvivo12, a widely recognized software for qualitative analysis. This methodological approach ensures a comprehensive and meticulous examination of the data, facilitating a deeper understanding of the phenomena under study.

## Data analysis

The interview recordings were meticulously transcribed and translated from Chinese to English. Each transcript was thoroughly reviewed to ensure the accuracy of the translation, grammatical integrity, and logical consistency. For the analysis, we utilized Nvivo12, a widely recognized qualitative analysis software, and conducted thematic analysis to identify patterns, similarities, and differences, thereby extracting relevant themes (Ryan and Bernard, 2003). The lead researcher focused on coding the following aspects: (1) the disembedding experiences of divorced Chinese youth; (2) the dynamics of their online social interactions; and (3) the transformative life changes following their re-embedding. To ensure the reliability and validity of our analysis and interpretations, we implemented inter-coder reliability checks (Lavrakas, 2008). This involved multiple coders to confirm the consistency of the coding framework and ensure that the identified themes aligned accurately with the original data.

### Escaping reality: exploring the disembedding practices of divorced Chinese youth

Divorced Chinese youth, confronted by the pressures of their current circumstances, the enduring impact of past marriages, and difficulties in navigating face-to-face social interactions, discover that their fundamental needs remain unmet. As a coping mechanism, they resort to “disembedding,” temporarily withdrawing from these challenges by transitioning from physical to digital realms. Despite the disembodied nature of information exchange and the absence of physical presence, the authenticity and recognition of each individual’s identity persist, without nullifying the prevailing power dynamics within the realm of information exchange.

In China, divorce frequently results in stigmatization, marking those who are divorced with a sense of dishonor (Fu and Wang, 2019). This sentiment is echoed in an ancient Chinese proverb shared by S16: “You should not air your dirty laundry in public.” This stereotype persistently undermines the psychological well-being of divorced young adults in China, leaving them unable to withstand societal prejudice. To cope, they seek emotional solace and new social connections by distancing themselves from their physical surroundings, a process termed “passive disembedding.” Throughout this experience, divorced young adults in China often articulate feelings of dissatisfaction and resistance. As expressed by S16:

I don't believe divorce should be used to categorize a group. Why designate it as a “divorced group?” Some individuals may not be officially divorced, yet they experience strained relationships. I contend that they too undergo psychological divorce.

S16 expresses dissatisfaction with this categorization, offering a critique of societal labeling practices. The term “divorced” has evolved into a stigmatizing label for these Chinese youth, marking them as if symbolizing social inferiority. Unlike their never-married peers, divorced individuals often face marginalization in both the marriage market and wider social interactions, frequently being viewed as “devalued.” This perception has gradually crystallized into a societal consensus, firmly ingrained in collective consciousness. As articulated by S8 to the researcher:

In a neighborhood WeChat group, I openly mentioned my divorce as I've always been forthright about it. However, a woman in the group retorted, “You're divorced and you still have the audacity to discuss it.” In that instant, I realized they likely perceive divorce as shameful, indicative of failure, and a symbol of inadequacy.

The discourse within the group chat reflects a microcosm of the broader Chinese societal attitude toward divorced individuals, embodying an “emotional habitus” that combines personal subjectivity with societal objectivity. This interplay influences individual behaviors, revealing prevalent stereotypes associated with divorce. Moreover, the hierarchical nature of Chinese social relationships imposes additional real-life pressures on divorced Chinese youth, particularly within close-knit social circles, as articulated by S15:

My family speaks in a very hurtful way, suggesting that once you're divorced, no one will want you. They say, “Think about how pitiful the child is, it's all because of you. Now he won't have a mother,” and they lay all these burdens on me. It's a form of psychological torture, constantly instilling in me the belief that being divorced means being unwanted.

In traditional Chinese culture, divorce is often regarded as dishonorable, sometimes synonymous with a failed life. This perception persists even in China’s more individualistic urban areas, where divorce decisions extend beyond personal choice, entwined with broader family and societal considerations (Yan, 2013). The Chinese adage, “A harmonious family leads to prosperity in everything,” underscores the belief that marriage not only unites individuals but also intertwines families, influencing life quality, happiness, and familial harmony. Additionally, WeChat, one of China’s leading social media platforms, serves as a conduit for intimate real-life connections. However, self-presentation in digital spaces is often scrutinized and shaped by the expectations of one’s familiar societal circle. S12 opines:

Actually, the intentions of friends and family might be good, but I feel overwhelmed by this excessive concern and find it hard to adapt. Sometimes, I just try to avoid socializing on WeChat.

Research indicates that parents, particularly those with only one child, wield significant influence over their offspring’s marital affairs (Yan, 2013). This dynamic is exemplified in S12’s narrative, which underscores the deeply ingrained biases within traditional ideologies against divorced individuals. It elucidates how even close friends and family may lack a comprehensive understanding of their challenges. Consequently, such prejudices compel divorced youth to conceal their personal struggles from those closest to them. In the Chinese cultural context, preserving “face” or social status is paramount. To circumvent the discomfort and humiliation associated with losing face, divorced Chinese youth often retreat from their established social networks, seeking solace and impartial support from unfamiliar communities. In the internet age, there is a departure from Giddens’ concept of “disembedding.” The digital “disembedding” observed does not sever previous social relationships; rather, it offers respite from the distress of offline interactions through the realm of cyberspace.

The narratives of divorced Chinese youth exhibit diversity, yet interviews consistently reveal shared traumatic experiences, with the primary reasons for divorce often revolving around moral and legal transgressions, including instances of domestic violence (S15), infidelity (S17), and conflicts between mothers-in-law and daughters-in-law (S6). In China, tensions between these familial roles are particularly pronounced, frequently serving as catalysts for marital discord. The husband’s ability to navigate these relationships is pivotal, and a lack of effective communication can contribute to the dissolution of marital ties.

Post-divorce individuals without children often harbor aspirations to have offspring, thereby intensifying the desire for a new partner (Lampard and Peggs, 1999). Among the divorced youth partaking in this study, the majority are already parents, presenting a contrast in experiences compared to their childless counterparts. S15 articulated concerns regarding future childbearing:

In my situation, it's going to be difficult to find a partner because I might not plan on having more children. I believe many people with a history of divorce don't have the desire to have more children. However, many people in their 30s are very eager to have children, which might make me seem incompatible.

Divorced youth with children may resist the idea of expanding their families due to emotional, physical, and psychological factors. The crux of the issue often lies in the differing reproductive goals between prospective partners, leading to avoidance behaviors in relationships. Such dynamics not only narrow down the pool of potential partners for divorced youth but also influence their social interactions. At a more profound level, divorced youth commonly harbor psychological apprehensions about entering into another marriage, fearing it may exacerbate their emotional distress. This sentiment is succinctly captured in the reflections of S15:

Regarding remarriage, there is absolutely no urgency. I've found that many divorced individuals are quite cautious about remarriage. Unlike older, never-married groups who are eager to find someone with marriage in mind, divorced individuals do not share this urgency. Instead, suitability remains the primary consideration.

“Suitability” has emerged as a pivotal criterion for divorced individuals in their quest for a new partner, in stark contrast to the priorities of older, never-married individuals. Furthermore, owing to a shrinking social circle and prevailing societal biases, individuals often eschew seeking relationships within their immediate surroundings, opting instead for online avenues. This deliberate detachment is characterized as “purposeful disembedding.” According to S15, those with prior marriage experience typically prioritize practical considerations. Having navigated marriage previously, they tend to undergo both physical and psychological transformations, fostering greater independence, as underscored by both S13 and S15. Hetherington (2003), drawing upon longitudinal data, noted that divorcees initially contend with varying degrees of physical and emotional distress; however, most eventually adapt to their new circumstances, though a subset continues to wrestle with lingering anguish. Reflecting on the adverse effects, divorce also engendered a sense of diminished confidence in S2:

The biggest change in mindset might be a loss of confidence when seeking relationships after a divorce, as having had a marriage before tends to be a concern for many. I feel that women, in this situation, might experience an even greater loss of confidence compared to men. To be frank, it seems that men are generally more sensitive to issues like being previously married or divorced, especially if a woman has a child. Many men, I perceive, are particularly troubled by the idea of dating women who are divorced and have children.

The lack of self-confidence among divorced young adults in China highlights their inclination to express emotions and seek resonance through the anonymity of cyberspace. Beyond merely indicating a lack of confidence, S2 also illuminates gender-based disparities in post-divorce mindsets, reflecting China’s traditional societal norms where “men are expected to work outside, while women are tasked with household management.” This cultural expectation results in women devoting more time to familial duties within the marriage, while men focus on their careers, fostering an imbalanced family dynamic that leads to a discrepancy in status between genders post-divorce.

Marx posited, “The essence of man is not an abstraction inherent in each single individual. In reality, it is the ensemble of social relations.” Seeking emotional solace and support, divorced youth engage in social interactions through three primary avenues: seeking assistance from relatives and friends, participating in offline matchmaking, and engaging in offline dating. However, even when interacting with relatives and friends, divorced youth often hesitate to share their deepest concerns, finding it challenging to fully alleviate their feelings of distress and helplessness. S13 shared with the researcher:

I can’t talk too much with my family because they worry and are anxious, and I'm not young anymore, so I feel somewhat embarrassed to go back and discuss it with them. Sometimes I talk about these issues with colleagues at work or other friends, but they have their own families.

In contrast to their married counterparts, divorced individuals frequently endure heightened loneliness and diminished satisfaction with their social lives. While divorced youth may attempt to alleviate these negative sentiments through interactions with relatives and friends, the narratives of the interviewees indicate that underlying, suppressed emotions persist unattended. Essentially, the internal struggle to uphold social prestige (“face”) and the practicalities of their external circumstances pose formidable challenges to effective reconciliation. S14 expressed his sentiments:

If I share some of my unpleasant experiences with friends, I worry that they might first suffer a negative impact psychologically.

Divorced youth frequently find it challenging to secure emotional support from those nearest to them. While friends and parents are often deemed the most reliable confidants, the traditional Chinese value of “filial piety” leads many children to adopt a “report the good but not the bad” approach with their parents, aiming to shield them from worry. S17 shared her approach at the time and her parents’ attitude toward divorce:

My parents are those traditional Chinese parents from the countryside, very down-to-earth and honest, a hardworking rural couple. They couldn’t understand why we would divorce over a lack of love. In their view, once children are involved and life can go on, it should go on, regardless of the difficulties.

Divorce not only dissolves the marital union but also often disrupts relationships with mutual friends, potentially endangering long-standing connections. Loyalty dilemmas frequently cause mutual friends to distance themselves post-divorce, hindering their ability to stay in touch with both parties. When divorced youth find themselves unable to derive emotional support from their connections with relatives and friends, the challenges of their real-life situations drive them to disengage and seek new connections in digital realms. The anonymity and superficial connections characteristic of online social networks cater to the needs of divorced youth, offering them a platform to freely express themselves.

### “Socializing in the clouds”: “re-embedding” in cyberspace

The process of re-embedding can paradoxically serve as a source of alienation; re-engaging with new structures often weakens existing relationships. As conditions evolve, re-embedding disrupts traditional connections, yet it simultaneously empowers divorced young adults to actively seek new references for constructing their identities. Cyberspace has emerged as a refuge for divorced youth, who disengage from their real-life environments and re-embed themselves into new domains. This spatial transition is often marked by feelings of passivity and resignation, along with insecurity and uncertainty. This uncertainty stems from the unstable fluidity characteristic of modern society’s transition from disembedding to re-embedding (Lee, 2011).

In our daily interactions, we assume the role of “actors,” navigating the need to present various facets of ourselves to different audiences while aiming to maintain consistency and stability in our self-presentation. Goffman argues that this balancing act is fraught with the constant risk of failing to sustain our desired self-image. Research indicates that individuals are more likely to divulge personal information in online settings—information they would typically conceal in face-to-face interactions—thereby shedding their social “masks” (Bargh et al., 2002). However, within the domain of social media, particularly in interactions with the opposite sex, divorced youth often struggle to express their authentic selves, even in relationships characterized by weak ties. The act of curating one’s identity on social media can be likened to producing a personal narrative film, in which experiences of divorce are edited out like blemished memories. By strategically concealing certain aspects of their lives, divorced youth are able to prevent the collapse of their impression management efforts. S3 articulates their approach to marital status through the concepts of “privacy” and “deduction”:

Firstly, I consider this a matter of privacy, which I value significantly. On another note, I see it as a detracting issue because, in China, families with a history of divorce might be perceived as problematic.

Societal stereotypes concerning divorced youth significantly influence their self-expression online, prompting a cautious approach to how they present themselves on social platforms. Despite this cautiousness, there remains a compelling need for these individuals to disclose their genuine circumstances when circumstances necessitate transparency. S2 elucidated this phenomenon by sharing his experience:

If we get along, I’ll typically disclose my divorced status when the topic of marital status comes up. However, if the conversation fizzles out after a few exchanges, then I don’t see the need to mention that I’m divorced.

The cautious stance adopted by divorced youth can be interpreted as both a mechanism to safeguard privacy and a defense against prevailing stereotypes. This approach also reflects a broader trend of diminished intimacy in social interactions, a phenomenon not unique to divorced individuals but observed across various social contexts.

Dating apps have become increasingly integral to the social lives of young individuals, offering a convenient method to connect with potential partners nearby (Newett et al., 2018). This geolocation-based matching mechanism functions logically through algorithmic operations designed to categorize and filter compatible individuals from a data pool for users (Sharabi, 2021). Typically, the operational mode of these apps enables users to indicate their preferences, such as liking or choosing to match with someone, through the user interface (Hu and Wang, 2023). S11 describes their experience with this technology as follows:

On Soul’s homepage, there is an interpersonal interaction graph. If you find someone interesting, you can click to directly access their profile, where some people also post things like their own marriage resumes. If you are interested, you can either private message them or follow them. Subsequently, your interactions with them will display a compatibility score on your homepage, which is generally based on various personality tags to assess the degree of match.

Existing research, such as Illouz (2007), posits that love cannot be quantified through calculations and algorithms; nonetheless, our findings indicate that algorithmic recommendations on dating apps offer divorced young adults in China a convenient matching process. This facilitates rapid connection with suitable conversation partners, thereby implicitly enhancing the likelihood of successful matches. Our observations suggest that these individuals often place significant trust in these algorithmic recommendations, primarily because the specific goals of divorced young adults in China align closely with the precision of algorithmic filtering.

Contrary to expectations that divorced youth might predominantly use these platforms to seek new spouses, our empirical data reveals a marked divergence. Many interviewees expressed a disinterest in remarrying. Instead, they frequently utilize dating apps for emotional solace, creating a space where they can experience relief and the freedom to “breathe.” S12 reflects on this phenomenon by stating:

Having already experienced marriage once, I might not view it as importantly as before. I’m more inclined toward having an undisturbed, comfortable, and free lifestyle.

Divorced young adults often find solace in cyberspace, where “freedom” characterizes their new lifestyle. This notion of liberty is pivotal as users of dating apps pursue varied objectives, analogous to how a smoker’s intentions influence their smoking behaviors (Flesia et al., 2021a), or the way individuals seeking sexual partners on these platforms may engage in higher risk sexual behaviors (Flesia et al., 2021b). However, for divorced young adults in China, online interactions serve primarily as a refuge from the harsh realities of their lives. These engagements offer a temporary respite, allowing them to momentarily distance themselves from the adversities associated with their previous marriages. Participant S14 articulates this perspective by stating:

After the divorce, I feel there’s a kind of oppression or a sense of disparity within myself. Using these social networking apps, I find that interacting with others helps me forget some of the unpleasant experiences from my previous marriage.

In the context of Chinese ritual culture, marriage is considered a critical life event. Therefore, the dissolution of a marriage is often viewed as a mark of dishonor, which can foster self-doubt among individuals. Faced with the difficulties of addressing these issues in real life, S8 turns to the online world, seeking support from strangers who have experienced similar challenges:

I want to understand whether other people’s experiences with this matter are similar to mine, and figure out whether the issue lies more with myself or with the other party. I also want to delve deeper into understanding the issues between men and women, the problems within marriage, and issues involving children.

Among the interviewees, a prevalent narrative exists where divorced youth create supportive networks. These networks facilitate the sharing of marital dilemmas and provide comfort through mutual understanding of each other’s personal struggles, thereby enhancing community cohesion. This behavior reflects the broader concept of homophily observed on dating platforms, where individuals tend to connect with others who share similarities in appearance, race, educational background, and other attributes (Harrison and Saeed, 1977). Consequently, participants, including S8, achieve a deeper understanding of each other’s communicative goals:

On the surface, he might describe his situation very simply or casually, but in reality, his expectations or demands for a partner might be higher.

Haunschild (2004) expanded the concept of re-embedding by introducing the term “economic re-embeddedness,” suggesting that individuals consider resource exchange relationships during the re-embedding process. S8’s account illuminates the underlying motivations why divorced youth increasingly turn to dating apps for online engagement. Their apprehension regarding remarriage has evolved into a “disappearance of sentimentality” and a “rational reconstruction” of their approach to relationships. Rather than rushing into another marriage, they opt to relish the social interactions provided by dating apps, which are notably lacking in their offline work and personal environments. S10 articulated this perspective to the researcher as follows:

I feel that online interactions expose me to a more diverse group of people. In my immediate surroundings, I tend to encounter individuals who are similar to me, particularly those in the legal profession.

Divorced youth confront two principal social challenges: the demanding nature of their professional lives and the inflexibility of their established social networks. The accessibility and diversity provided by online environments are well-suited to address these specific issues. As articulated by S7:

I think offline, after taking care of the children, I might be too exhausted and just want to relax and chat on my phone.

The interaction model within internet spaces presents distinct challenges for divorced youth. An ancient Chinese proverb declares, “At 30, one should stand firm,” implying that by this age, individuals are expected to have achieved significant life milestones. Many divorced individuals within this age group face both life adversities and occupational stress. Their perception of time sharply contrasts with the fleeting nature of today’s online social interactions, which are often compared to “fast food.” In the current context, the pursuit of “security” in relationships frequently erodes the spontaneity of love, leading to its rationalization as decisions are increasingly driven by pragmatic considerations. S10 articulated these sentiments by stating:

I feel that on these social networking apps, there might be some people with whom you do not engage in long, in-depth conversations. I think this can be quite a waste of time.

Secondly, the geolocation feature of dating apps, which facilitates interactions based on user proximity, raises concerns among divorced youth. While superficially, their apprehension centers on “encountering acquaintances,” at a deeper level, it reflects a desire to maintain privacy regarding their divorced status to avoid unwanted public exposure. As a result, the nature of intimate connections on social platforms has transitioned into forms of mediated, networked, or public intimacy (Chambers, 2013, p. 164; De Ridder, 2015; Miguel, 2018, pp. 26, 27). From another perspective, this cautious approach can also be seen as a means of self-protection against societal stigma. S12 articulated this perspective by stating:

The main concern is actually about interacting with the opposite sex because sometimes I think about the possibility of offline encounters in the same city. I'm a bit worried about overlaps with my social circle, and the fact that I’m divorced might become known to her.

Consequently, the prevailing social norms in China do not entirely absolve divorced youth, who often face “the gaze of the other” during social interactions. This scrutiny significantly reduces their willingness to engage actively in socializing, prompting them to seek temporary “dignity” by concealing their true selves. S3 observed the following phenomena:

Some people view being divorced as a negative factor because, in China, families with a history of divorce are often thought to have certain issues. As a result, some individuals are hesitant to form close relationships with those who have such experiences.

Although dating apps are often perceived as empowering for women, providing them with various “privileges,” they inadvertently obscure deep-seated gender inequalities (Scarcelli et al., 2021). These disparities are rooted in societal double standards concerning gender roles, marital expectations, and state policies. Single mothers, in particular, face heightened discrimination, primarily due to the intense moral scrutiny directed at women in society. S7 described this societal reality as follows:

But when it comes to the opposite sex, I might take this into consideration because some men have prejudices against single mothers. For instance, at work, I might face cold treatment from others.

In China, festivals such as the Mid-Autumn Festival, Spring Festival, and Lantern Festival symbolize “reunion,” a theme that underscores togetherness. This emphasis can exacerbate feelings of loneliness among divorced youth who have recently experienced the dissolution of their marriages, leading to heightened social anxiety during these culturally significant periods. S7 elaborated on this phenomenon, noting:

Sometimes I do feel a strong sense of urgency, especially during periods like the New Year. Seeing relatives and friends together as a family of three or four can make me feel particularly eager.

In Chinese society, emotions are not solely personal experiences but are deeply entangled with socio-economic frameworks and power dynamics. As Beckert (2007) argues, embeddedness is a multifaceted concept that encompasses various dimensions including cultural, political, and cognitive aspects. Consequently, the most profound expressions of emotion often surface in the everyday lives of ordinary individuals. This phenomenon reflects the ambivalence experienced by divorced youth who navigate the dual desires for companionship and aspirations for a renewed existence. As S9 mentioned:

Actually, I don’t want to keep searching like this. I just want to settle down, live a good life, and work well. I don’t want to keep drifting because I think the process of searching is quite torturous for a girl, maybe because I’m more of a traditional girl.

Throughout this arduous process, the emotional “reserves” of divorced youth are substantially depleted, leaving them feeling drained. Each attempt to engage in social interactions demands considerable courage, and the resultant pain frequently shatters their remaining defenses. As S9 mentioned:

I feel like my passion is quickly running out, you know? Because you have to sort out your feelings, and I'm not the type to play games with emotions, so I take every relationship seriously. Plus, everyone asks why you got divorced, and then you have to talk about a part of your past that isn’t really happy. I feel like it’s draining for me.

### Post-re-embedding: examining the impact of group companionship on life outcomes

Upon integrating into cyberspace, divorced youth derive social support and emotional comfort from participating in various group activities within this digital environment. The continuous positive reinforcement they encounter online empowers them to address real-life challenges and mitigate the complex pressures associated with divorce. As these individuals invest more effort into these digital relationships, the connections become stronger and more profound, fostering trust, a sense of belonging, and mutual support among them.

Divorced youth increasingly turn to cyberspace as a refuge, where they encounter social support in this virtual sanctuary. This observation aligns with findings that suggest an increase in the number of friends following divorce, along with a phase of online “expansion.” The networks formed during this period can be either transient or enduring (Albeck and Kaydar, 2002). Despite these positive developments, societal attitudes remain harsh, often labeling them with stigmatizing terms such as “the divorced group,” “divorced individuals,” “divorcees,” and “those in second marriages.” As S16 stated:

Not having children essentially means it was as if I just had a romantic relationship.

S2 is resolutely committed to overcoming conventional prejudices and is actively seeking to discard these “labels” in pursuit of a normalized life:

Because I think defining people who have gone through certain experiences as a group actually exaggerates the impact of those experiences.

Labeling adversely affects the enthusiasm of divorced youth for social interactions, causing them to subconsciously distance themselves from individuals who are not divorced, whom they view as perpetuators of stereotypes. S13 recounted a relevant experience:

If someone is unmarried, I’m also reluctant to talk to them because they might think being divorced, as if going into a second marriage, somehow makes you less valuable. I just feel like not bothering with them, and I don’t think there’s any need to explain anything to them.

S13’s statement underscores that although divorced youth may lack the power to change societal prejudices, they are able to mitigate secondary harm through personal resistance. S7 further elaborated on this issue:

People who are divorced have experienced emotional trauma, or rather, they are able to provide guidance and help me.

From a social networking perspective, divorced youth frequently seek support among their peers, sharing experiences and building emotional bonds. However, a portion of these individuals exhibits reluctance to engage with fellow divorcees, instead showing a preference for the company of lively, unmarried young adults. S15 elaborated on this phenomenon, stating:

Actually, talking to divorced individuals feels like conversing with plain water, but with younger people, it’s more like conversing with beverage.

The metaphors “plain water” and “beverage” are used to symbolize “boring” and “interesting,” respectively. This conceptualization is corroborated by other interviewees, such as S12, who asserts:

Young people tend to be very active in posting on their social circles, whereas those of us in our thirties and forties have social circles that are very quiet, or rather, very homogeneous, dull, and uninteresting.

This realization has prompted S12 to engage in deeper contemplation, leading to the conclusion that divorced youth often construct a semblance of emotional stability. Viewed from another angle, this behavior could be interpreted as indicative of life’s journey. Consequently, it becomes clear that despite the diversity in their social circles, divorced youth share a common pursuit of understanding, companionship, and empathy. They aspire for societal acceptance, striving to overcome prevailing biases and reintegrate into everyday life. Therefore, they are not anomalies but ordinary individuals navigating their unique circumstances. S14 articulated this perspective, stating:

Interacting online makes me feel like I’m not alone in society. It gives me a sense of companionship and keeps me from feeling extremely isolated and cold.

Upon integrating into cyberspace, divorced youth experience significant transformations in their lifestyles, methods of interaction, and psychological states. In the contemporary digital era, unlike in times when the internet was less pervasive, dating apps now function as sanctuaries for these individuals. Online engagements have notably alleviated the somberness and melancholy associated with divorce, expanded their social networks, and facilitated encounters with new “strangers.” Previous research has shown that divorced individuals feel more connected to their personal networks and participate in a greater number of social activities than before their divorce (Thuen and Eikeland, 1998). As S11 expressed:

I think that through online interactions, there are some common chat rooms. Meeting some friends, both male and female, in these chat rooms, I believe, also enriches my circle of online friends.

Online social support exerts a positive impact on the mindset of divorced youth, fostering greater openness and ease in self-expression. This subtle form of comfort, metaphorically described as “moistening everything silently,” often suffices to dispel the lingering shadows in their hearts. What may appear to others as minor support and companionship assumes a significant role for these individuals, akin to “fuel delivered in the snow,” providing essential sustenance and warmth during challenging times. S14 believes:

I’ve found a particularly good advantage online: you definitely won’t have social anxiety. As for life and work, I think it acts like a seasoning, not bringing earth-shattering changes, but I believe it can provide a kind of comfort or relief to my mind.

An interviewee recounted her experiences, vividly detailing the obstacles she faced in private social interactions and underscoring the reasons why divorced youth prefer online engagements. S17 elaborated on her motivations, stating:

The pressure is immense when you reach middle age. Sometimes you just want to post something on your social circle, but you might be spreading negative energy to others.

In China’s relational society, divorced youth are often compelled to consider their peers’ perspectives, which can significantly influence their career progression, business dealings, and relationships with friends and family. Consequently, they frequently channel the emotional repercussions of divorce through online platforms. This phenomenon was highlighted by S2, who noted:

Meeting new people also adds a bit of fun to my life.

The enjoyment derived from these interactions is primarily attributed to positive communication. S16 prefers not to dwell on or revisit the past:

Because you know that some things are in the past and shouldn’t be brought up again. If we revisit the topic of divorce, it feels like touching a scar.

As the internet transcends time and space, individuals often find themselves anchored in a single location, replaying their historical memories (Castells, 1997, p. 6–7). To circumvent the continual revisitation of their divorce experiences, interviewee S9 proposed the following strategy:

I’ve essentially written a short essay about it, so when someone asks, I just send it to them. I don’t want to have to recount my divorce experience all over again.

For divorced youth, online interactions have the potential to transform social dynamics, thereby restoring their confidence in life following the disappointment of a failed marriage. However, realizing this transformation and its positive effects requires them to continuously adapt their interaction strategies and habits. This adaptation is essential to prevent further emotional distress.

## Conclusion and discussion

Social and service platforms across various sectors have become increasingly essential for the smooth functioning of daily life, immersing individuals into a “Platform Society” characterized by layers of new protocols (Van Dijck et al., 2018). Digital platforms provide new modes of coexistence, reflecting the multi-spatial and multi-attribute existence that mirrors the modern human condition. As Manuel Castells points out, the network society forms a new sociotemporal reality, where individuals, once isolated, discover a public space that reconnects them. This space of mass communication, mediated by internet social networks, enables individuals to exchange opinions and share perspectives in this communal realm (Castells, 2015, pp. 1, 2). Nonetheless, our findings reveal that some divorced young adults prefer to withhold their location information more so than older singles, due to concerns about being recognized by local friends and relatives offline. This suggests that even in the digital realm, the processes of “disembedding” and “re-embedding” cannot fully mitigate the algorithm-driven convergence of online and offline spaces.

This shift heralds the creation of multiple spaces where digital platforms enable novel forms of coexistence, reflecting the complex, multi-spatial, and multifaceted reality of contemporary life. Current research demonstrates that the online interactions of divorced youth in China involve a dynamic process of “disembedding” and “re-embedding.” While these interactions share similarities with those of the broader demographic of older singles, the real-life experiences and interaction patterns of divorced youth significantly diverge from those of never-married individuals, highlighting the interplay between real-world challenges and online engagement. In this study, we explore how divorced youth in China navigate their inner turmoil, seek relief from reality, and pursue emotional support and psychological solace. Although divorce is a global phenomenon, our findings reveal unique social and cultural dimensions within the Chinese context. Additionally, our focus on younger divorcees reflects the emerging trend of early-age divorces in China, providing insights into the distinct experiences of this demographic.

Firstly, divorced young adults in China frequently encounter stigmatization. Goffman (1963, p. 1) defines stigma as an attribute, either physical or moral, that degrades an individual from a whole to a tainted, diminished status. Our analysis suggests that this stigma originates from China’s deep-seated prejudices toward marital relationships, often perpetuated by the public, with parents and friends sometimes acting as the initiators of these biases. This underscores the importance of “face,” a crucial aspect of maintaining “guanxi” (relationships) in Chinese culture, even among relatives. Brown and Levinson (1987, p. 61) describe “face” as the positive social value a person claims for themselves through others’ perceptions during interactions. Loss of “face” can lead to psychological and behavioral distancing by others. Consequently, divorced young adults in China often become prime targets for social exclusion and labeling, as suggested by social psychology’s “labeling theory.” When an individual engages in what is considered “primary deviance,” those around them tend to label and reinterpret the individual’s past actions (Becker, 1963, pp. 7, 8). Moreover, this group finds themselves at a critical juncture, balancing career progression and interpersonal relationships. The breakdown of social networks post-divorce (Spanier and Casto, 1979) intensifies their anxiety and distress about their situation. Although friends and family typically offer support during times of pain or trauma, traditional norms may prevent divorced youth from fully disclosing their challenges, pushing them toward self-soothing measures for relief. Furthermore, the myriad pressures of daily life compel them to seek ways to alleviate this stress. It is common for divorced individuals to proactively manage their situation by re-establishing their social networks and redefining their social lives, effectively compensating for the loss of a partner (Gerstel, 1988). This research illuminates the coping mechanisms and self-adjustment challenges faced by divorced youth in China, rooted in the complex dynamics and societal contexts of Chinese culture.

**Figure 1:**
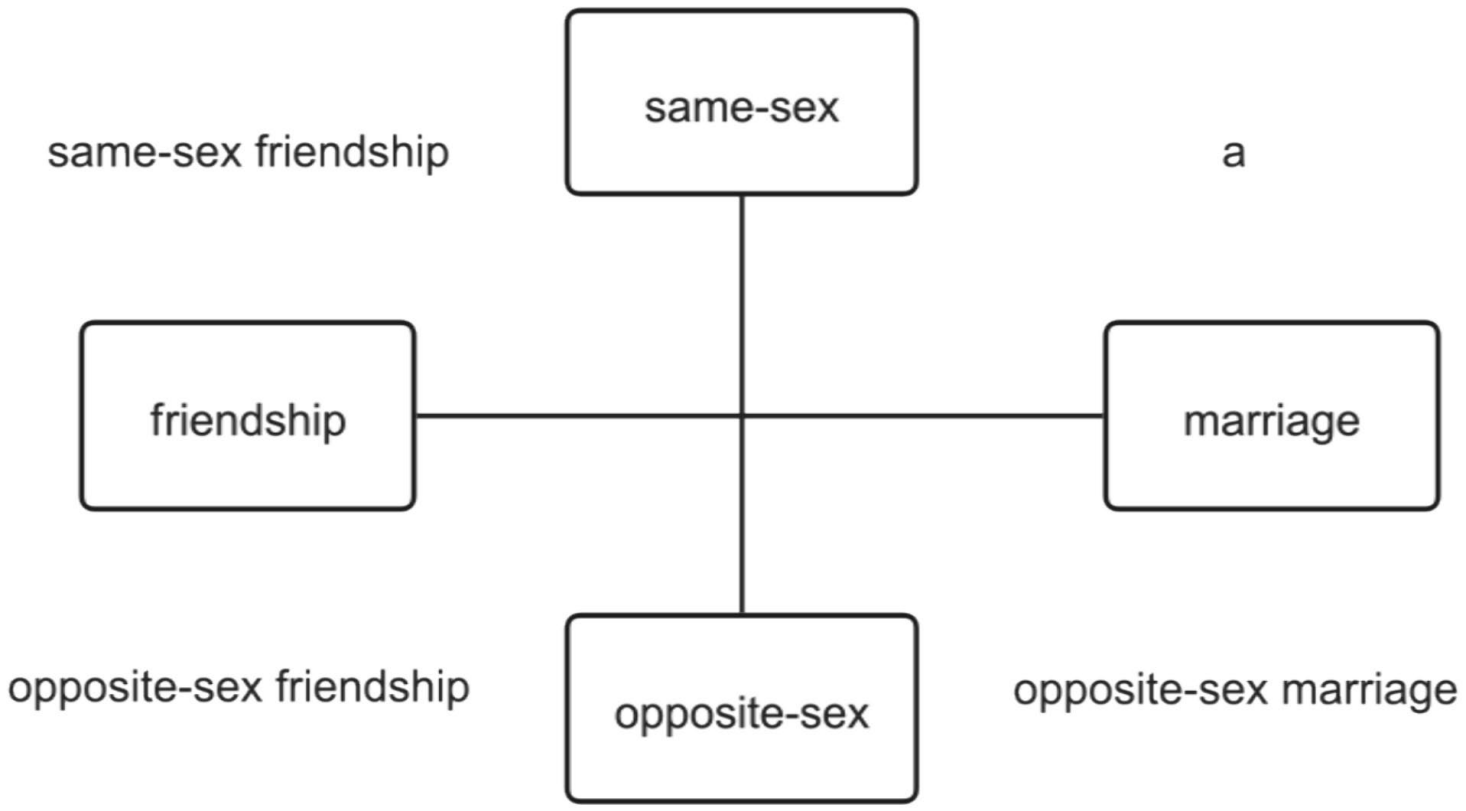
The online interaction objectives of divorced youth in China. ^a^Same-sex marriage has not yet been legally recognized in China.

Secondly, divorced young adults in China increasingly seek support and assistance in cyberspace to meet their specific needs. The Uses and Gratifications Theory highlights that individual motivations and behaviors are central to media use, positing that the realization and extent of need satisfaction, alongside subsequent effects, determine the outcomes of media usage (Ruggiero, 2000). Unlike typical motivations for using dating apps, such as seeking sexual partners (Lee, 2019) or marriage partners (Liu and Lin, 2023), Chinese divorced young adults often do not prioritize dating, and their interest in remarriage varies individually. Our research indicates that the interaction purposes of Chinese divorced young adults are multifaceted; thus, we have identified several objectives for their interactions (Figure 1): forming same-sex friendships, opposite-sex friendships, and seeking opposite-sex marriage. Notably, we have not observed instances of same-sex marriage, possibly reflecting China’s unique social and legal context. These interaction goals, while common, hold particular significance for divorced youth in China. Many express reluctance to remarry, and their approaches to forming new relationships vary. Some divorced young adults prefer to connect with others who are also divorced, seeking internal group support and companionship. Computer-mediated communication often surpasses face-to-face interactions in fostering emotional connections, as individuals who have never met can develop significant “social identity categorization” due to shared interests (Walther, 1996). In China, divorce marks a critical juncture in life, leaving many divorced young adults feeling confused and helpless. They seek solutions and life experiences from others with similar experiences. Previous studies have noted that middle age often brings multiple life changes, especially for women, and receiving social support during this period is likely crucial (Bresnahan and Murray-Johnson, 2002). Conversely, some divorced young adults are hesitant to engage with the divorced community, preferring to use dating apps to meet people from diverse backgrounds, professions, and personalities to avoid homogenized interactions. Based on their painful experiences, they aim to escape from a group marked by suppression and sorrow. Furthermore, interactions with the opposite sex are categorized into friendship and marriage. The latter often resembles a transactional, “commodity-style” selection process in an online “matchmaking market,” prioritizing clear intentions toward marriage to avoid the inefficiencies of casual dating. However, the absence of physical presence in these interactions has its limitations, as the need for physical intimacy cannot be indefinitely overlooked. Those who view themselves as unsuitable for remarriage often opt for platonic relationships, focusing on friendships with the opposite sex. Lingering negative emotions from divorce, coupled with potential disputes over life arrangements and custody, can diminish the desire to pursue new personal relationships (Jacobson, 1983). This analysis elucidates the varied online interaction goals of divorced youth in China, offering a comprehensive view of their digital engagement landscape and highlighting the nuanced ways they navigate their post-divorce lives online.

Finally, the social recommendation systems of dating apps restrict users’ autonomy by compelling them to select and utilize these platforms (Hu, 2023). However, our research indicates that divorced young adults in China, constrained by the demands of life and limited time for offline interactions, primarily opt for dating apps not out of necessity but as a preference. In an increasingly mediated and platform-driven world, users are often compelled to relinquish their choices to machines and algorithms (Rosenfeld et al., 2019). In this study, the necessity of transferring choice is evident, as the dating goals and filtering criteria of divorced young adults in China are specific, necessitating algorithmic recommendations to accurately target their desired social group. Dating apps utilize mobile device location systems to precisely identify nearby individuals and analyze personal information (De Ridder, 2022). However, we have found that some divorced youth do not wish to disclose their locations. Compared to older singles, they are more concerned about being discovered by local acquaintances offline. This indicates that in the internet environment, the processes of “disembedding” and “re-embedding” cannot shield against the algorithm-driven merging of online and offline spaces.

Our study is subject to several limitations. Firstly, it focuses exclusively on divorced youth in China, a demographic whose rising divorce rates highlight the relevance of our research. Future studies could expand the scope to include divorced individuals across various age groups, employing a life course perspective. Secondly, while gender differences may significantly affect the online behaviors, objectives, and experiences of divorced youth, our research did not extensively explore this dimension. This aspect deserves further exploration in future studies. Lastly, our methodology was limited to in-depth interviews, which did not include direct observation of online interactions among divorced youth in China. Future research could employ a variety of methodologies to obtain a more comprehensive understanding of the online engagement patterns of this demographic from multiple perspectives.

In conclusion, this study investigates the online interaction behaviors of divorced youth in China, illuminating their process of disengaging from real-life contexts and integrating into cyberspace. This exploration, addressing a gap in the existing literature, provides valuable insights for future research. Giddens’ concepts of disembedding and re-embedding effectively reflect the experiences of divorced youth in China, offering a framework through which to understand their life situations and strategies for online engagement. Driven by traditional Chinese marital norms, these individuals increasingly seek refuge in digital spaces, navigating the complexities of friendships and romantic relationships against the backdrop of conventional dating expectations. In the fluid landscape of the internet era, online interactions often manifest as fleeting encounters among numerous users, with minimal investment in deep connections. As some studies have suggested, the expansive reach of the internet raises individuals’ selection standards, promoting aspirations that extend beyond one’s immediate social circle (Sternberg, 1997). This environment may disadvantage divorced youth in China, potentially encouraging a more detached approach to remarriage.

## Data availability statement

The original contributions presented in the study are included in the article/supplementary material, further inquiries can be directed to the corresponding author.

## Ethics statement

Ethical approval was not required for the study involving humans in accordance with the local legislation and institutional requirements. The participants provided their written informed consent to participate in this study.

## Author contributions

JW: Writing – original draft, Writing – review & editing. JG: Formal analysis, Methodology, Writing – review & editing.
